# *Roupala montana* Aubl. Essential Oil: Chemical Composition and Emerging Biological Activities

**DOI:** 10.3390/molecules30163323

**Published:** 2025-08-08

**Authors:** Luis Cartuche, Mireya Guayllas-Avila, Leydy Nathaly Castillo, Vladimir Morocho

**Affiliations:** 1 Departamento de Química, Universidad Técnica Particular de Loja (UTPL), Calle París s/n y Praga, Loja 110107, Ecuador; lncastillo@utpl.edu.ec (L.N.C.); svmorocho@utpl.edu.ec (V.M.); 2 Carrera de Bioquímica y Farmacia, Universidad Técnica Particular de Loja (UTPL), Calle París s/n y Praga, Loja 110107, Ecuador; jmguayllas1@utpl.edu.ec

**Keywords:** *Roupala montana*, essential oil, phytol, acetylcholinesterase, enantioselectivity

## Abstract

*Roupala montana* (Proteaceae) is a shrub native to subtropical and tropical regions of Central and South America. The EO extracted from *R. montana* was analyzed for its chemical composition and biological activities. GC analysis revealed that the essential oil has a chemically diverse composition, predominantly composed of oxygenated diterpenes (29.37%) and sesquiterpene hydrocarbons (7.81%). Phytol, with 21.17 ± 1.59%, was the major component. Enantioselective GC showed a high enantiomeric excess of (S)-(+)-γ-muurolene (95.07%) and enantiomeric purity of (1S,4aR,8aR)-(−)-γ-cadinene. Antimicrobial, antifungal, and antioxidant properties were assessed in the EO, comparing them with related species, highlighting its potential for targeted pharmaceutical and biotechnological applications.

## 1. Introduction

Volatile secondary metabolites, commonly known as essential oils (EOs), are natural mixtures composed primarily of volatile organic molecules, such as monoterpenes and sesquiterpenes, that interfere with strains and bacteria, acting as antioxidant, cytotoxic, anti-inflammatory, and antimicrobial agents [1,2]. Because of the therapeutic potential of essential oils, exploring less-studied botanical families can lead to the discovery of novel compounds with promising pharmacological applications [3].

The botanical family Proteaceae contains more than 1700 species belonging to 83 genera distributed in Australia and South Africa [4]. In Ecuador, Proteaceae is represented by four native genera: *Roupala* (*Roupala cordifolia*, *Roupala monosperma*, and *Roupala obovate*), *Lomatia* (*Lomatia hirsuta*), *Oreocallis* (*Oreocallis grandiflora* and *Oreocallis mucronata*), and *Panopsis* (*Panopsis* Salisb), which are found in the Adean Forest [5].

**Figure 1:**
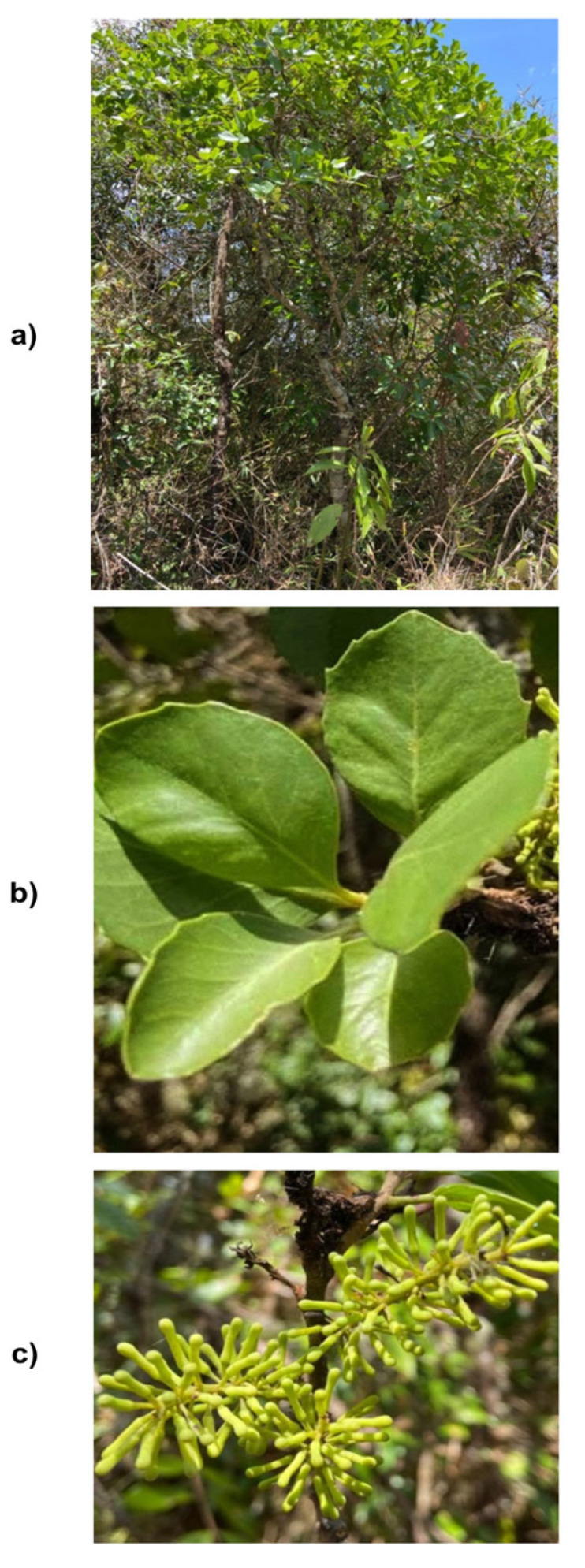
*Roupala montana*. (**a**) Tree; (**b**) leaves; (**c**) flowers.

*Roupala montana* is a tree that reaches heights between 6 and 20 m, with dimorphic leaves with toothed margins, 6 to 12 cm long, and an acute apex (Figure 1) [6]. 

Due to the unpleasant odor, like tuna, expelled by its bark and leaves, it is commonly known as “palo de cucaracha,” “palo muerto,” or “palo de zorrillo” [7]. Preliminary studies have described its biological activity, including antigenotoxic effects and protective activity against DNA damage, as well as the detection of flavonoids, such as quercetin and isorhamnetin, in its extracts, suggesting that the species may also exhibit antioxidant potential [8].

Although *Roupala* is considered one of the native genera of Proteaceae in Ecuador, *Roupala montana* has not been included in the documented species of the region. Only a study by Medina et al. has reported the presence of *R. montana* in southern Ecuador [9]. This limited evidence suggests that the species is either rare or underreported in national floristic records.

Therefore, the aim of this current investigation was to conduct a phytochemical study of the volatile constituents of the EO of *Roupala montana* to elucidate its chemical profile. In parallel, the EO was evaluated for potential antimicrobial, antioxidant, and anticholinesterase effects. This work seeks to contribute to the expanding literature of essential oils by providing novel insights into their therapeutic potential and possible industrial applications.

## 2. Results

### 2.1. Isolation of Essential Oil

A total of 100 g of the leaves of *Roupala montana* were distilled in three different batches to obtain a total of 0.10 mL of essential oil with an extraction yield of 0.10 ± 0.01% (*v*/*w*).

### 2.2. Chemical Constituents of Essential Oil

**Figure 2:**
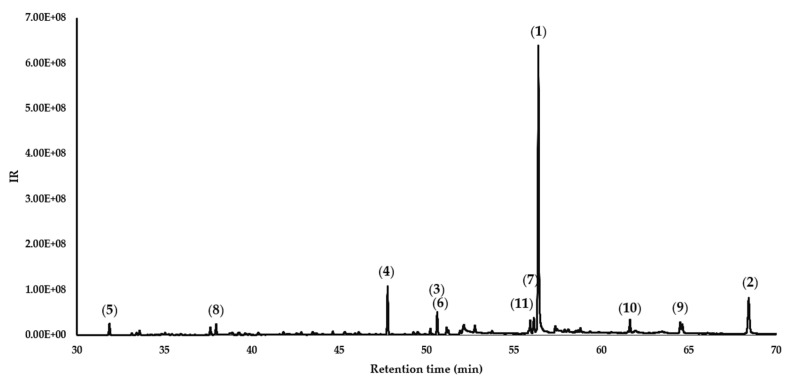
Gas chromatogram of *Roupala montana* leaf essential oil.

The gas chromatography (GC) analysis of the essential oil of *Roupala montana* revealed a complex profile, as illustrated in Figure 2 (see also, Supplementary Material Figure S1). This profile reveals sixty compounds identified and quantified as part of the *R*. *montana* essential oil. The overall composition was characterized by the presence of other compounds (55.21%), followed by oxygenated diterpenes (29.37%), while hydrocarbons and oxygenated hydrocarbon sesquiterpenes were found in lower abundances of about 6.49 and 7.81%, respectively. Finally, monoterpene hydrocarbons were the minor components occurring in the volatile fraction.

**Figure 3:**
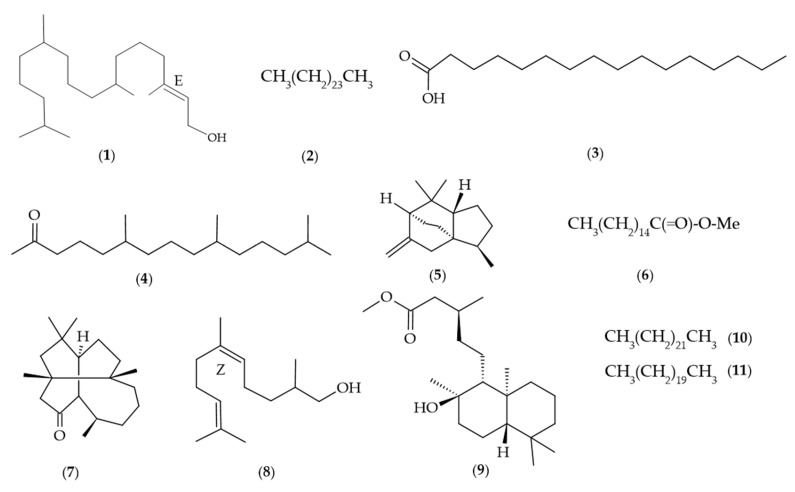
Main chemical compounds identified in *Roupala montana* EO.

Phytol represented the main component, with 21.17 ± 1.59 (**1**), followed by *n*-pentacosane (9.08 ± 0.04) (**2**), hexadecanoic acid (8.30 ± 0.77) (**3**), hexahydrofarnesyl acetone (6.28 ± 0.26) (**4**), *β*-duprezianene (3.16 ± 0.16) (**5**), methyl hexadecanoate (2.96 ± 0.12) (**6**), laurenan-2-one (2.68 ± 0.03) (**7**), (*Z*)-dihydro-apofarnesol (2.52 ± 0.10) (**8**), methyl labdanolate (2.46 ± 0.01) (**9**), *n*-tricosane (2.16 ± 0.00) (**10**), and *n*-heneicosane (2.00 ± 0.00) (**11**) (Figure 3).

A detailed analysis of the chemical composition of the EO of *R. montana* is presented in Table 1.

### 2.3. Enantiomeric Composition

Analysis of the *R. montana* essential oil using enantioselective GC columns showed that γ-muurolene existed as two enantiomers, with a significant enrichment of the (S)-(+) form (95.07% e.e.). In contrast, γ-cadinene was found to be enantiomerically pure, present exclusively as the (1S,4aR,8aR)-(−) isomer (Table 2, see also, Supplementary Material Figure S2).

### 2.4. Antifungal Activity

Table 3 shows the antifungal activity of the essential oil extracted from *R. montana* leaves. The assay demonstrated antifungal activity against *Aspergillus niger*. On the contrary, the EO did not exhibit inhibitory activity against *Candida albicans*, even at the maximum dose tested of 4000 ug/mL.

### 2.5. Antibacterial Activity

Table 4 details the MIC values of the volatile fraction extracted from *R. montana* leaves against a panel of bacterial strains. The EO demonstrated a lack of inhibitory activity against the Gram-positive cocci and most Gram-negative bacilli, with the exception of *Campylobacter jejuni*, with an MIC value of 500 ug/mL. Based on the data provided in the table, the EO from *Roupala montana* shows limited and selective antimicrobial activity.

### 2.6. Acetylcholinesterase Inhibitory Activity

**Figure 4:**
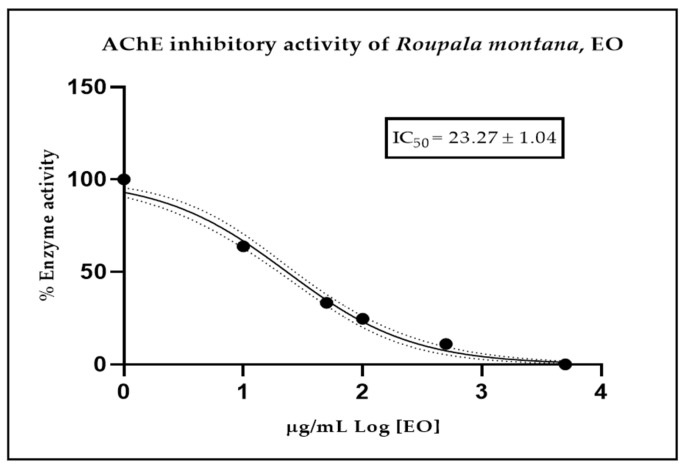
Half-maximum inhibitory concentration of *Roupala montana* EO against acetylcholinesterase enzyme.

Figure 4 provides a visual representation of the EO’s ability to inhibit the acetylcholinesterase enzyme. The IC_50_ value of 23.27 ± 1.04 µg/mL indicates a promising effect of the volatile fraction of *R*. *montana*. As a positive control, donepezil was employed, demonstrating an IC_50_ of 12.40 ± 1.35 µM.

### 2.7. Antioxidant Activity

The antioxidant capacity of *R. montana* was assessed using the ABTS and DPPH radical-scavenging assays, with Trolox serving as a positive control. Negligible antioxidant activity was observed for *R. montana* as the ABTS SC_50_ was below the detection limit at the highest tested concentration. Likewise, *R. montana* demonstrated no detectable antioxidant activity in the DPPH assay at the maximum tested concentration of 8 mg/mL. The results are presented in Table 5.

## 3. Discussion

The essential oil of *R. montana* mainly contains diterpenes, with phytol being the most abundant compound, representing 21.17 ± 1.59% of the total composition. However, a significant proportion of unclassified compounds contributes to the variability observed in the essential oil composition. In comparison, Medina et al. identified 15 compounds from the essential oil of *R. montana*, where the major components were kaur-16-ene (77.2%), kaur-15-ene (4.1%), and phytol (3.45%) [9]. These findings are partially consistent with our results, considering phytol as a major component of the volatile fraction.

In comparison, *Oreocallis grandiflora* (Proteaceae) essential oil has a chemical composition mainly composed of aromatic compounds (57%), followed by monoterpene hydrocarbons (22.5%) and oxygenated monoterpenes (15.4%). Key components include methyl eugenol (35.3%), α-terpinene (9.4%), *p*-cymene (8.3%), (*E*)-methyl isoeugenol (7.7%), and 1,8-cineol (7%) [13].

While the present study provides a comprehensive analysis of the essential oil composition of *Roupala montana*, comparisons with previously published phytochemical data are challenging due to the lack of research focused on the essential oil. To provide some context for our findings, we include comparisons with non-volatile fraction studies, acknowledging the inherent differences in phytochemical composition between essential oils and solvent extracts. It is important to emphasize that this comparison is made in the absence of more directly comparable essential oil data. As follows, alternative investigations have directed their focus toward the non-volatile fraction, such as a study conducted by Cunha et al., who reported the chemical constituents of the *n*-hexane fractions of the aerial parts of *R. montana*, wherein phytol (17.91%), γ-tocopherol (11.71%), ethyl pentadecanoate (8.89%), lupeol (8.05%), squalene (4.18%), β-amyrin (1.34%), *n*-tetracontane (3.85%), and α-amyrin (0.75%) were identified [14]. Furthermore, kaur-16-ene, linoleic acid, and α-tocopherol were isolated from the hexane extract of the leaves of *R. montana* [9].

Based on our knowledge, this study presents the first enantioselective analysis of *Roupala montana* essential oil. Regarding chiral or optically active constituents, scientific investigations have demonstrated that enantiomeric purity is essential to ensure the desired biological activity of a compound [15]. This holds significant relevance in fields such as pharmaceuticals, where the safety and efficacy of drug candidates are critical [16,17,18]. In *R. montana*, (S)-(+)-γ-muurolene and (1S,4aR,8aR)-(−)-γ-cadinene exhibit high enantiomeric purity within the essential oil for these specific compounds.

The biological activity of essential oils is generally understood to be primarily governed by their major constituents, influenced by the concentrations of these compounds and the modulating effects of minor components [19]. Species within the Proteaceae family demonstrate significant bioactive potential [20]. Phytol, a diterpene belonging to the class of long-chain unsaturated acyclic alcohols biosynthesized by nearly all photosynthetic organisms, possesses multiple bioactivities [21,22]. It finds application in cosmetics to mitigate oxidative stress-induced cellular senescence in keratinocytes and also exhibits antioxidant, anti-inflammatory, antimicrobial, cytotoxic, and immunomodulatory properties [17,23].

In this investigation, numerous assays were performed to determine the biological activity profile of *R. montana*. Antifungal activity against *Aspergillus niger* was observed, yielding a generally low value of 1000 µg/mL. *R. montana* EO demonstrated inefficacy against *Candida albicans*, suggesting either the absence of active compounds or their presence at sub-inhibitory concentrations against this specific yeast. Prior studies have focused on the biological properties of other fractions. Fungal inhibition by *R. montana* extract was evaluated against the *Trametes versicolor* and *Rhodonia placenta* pathogens, revealing that the *Roupala montana* extract inhibited the growth of *Rhodonia placenta* by approximately 80% and *Trametes versicolor* by 90% across all tested concentrations [24]. Similarly, antifungal activity was assessed in *R. montana* wood residues, demonstrating activity against *Cryptococcus neoformans* and *Cryptococcus gattii* but showing no effect against *Candida albicans* [25]. *Roupala braziliensis*, a variety of *R. montana*, exhibits significant variability in the efficacy of its extracts and fractions against diverse fungal strains. It demonstrated potent activity against *C. glabrata*, with an MIC of 15.6 µg/mL, indicating high selectivity toward this specific strain. It also displayed promising activity against *C. glabrata* and *C. krusei*, with MICs of 15.6 and 31.3 µg/mL, respectively. However, the activity against *Candida albicans* was moderate, ranging from 250 to 500 µg/mL, while the activity against *C. parapsilosis*, *C. tropicalis*, and *Cryptococcus neoformans* was generally low, exceeding 1000 µg/mL in many instances [26].

Regarding antibacterial potential, the essential oil of *Roupala montana* displayed limited and selective activity against the tested bacterial strains. Inhibition was observed only against *Campylobacter jejuni*, with an MIC of 500 µg/mL. Certain Proteaceae species could be compared with our study, such as the fruits of *Persoonia peniflora* R. Br. Moncayo-Molina et al. reported that the essential oil of *O. grandiflora* exhibited inhibitory activity against *Escherichia coli* and *Salmonella enterica*, with MICs of 8.32 ± 0.12 and 11.46 ± 0.12 µL/mL, respectively, indicating a moderate antibacterial effect [13]. In contrast, the lack of antimicrobial activity of *R. montana* EO suggests it is less effective than *O. grandiflora*, possibly due to differences in the concentration or nature of active constituents.

The extract of *p. peniflora* exhibited activity against the Gram-positive bacterium *Bacillus subtilis*, the Gram-negative bacterium *Escherichia coli*, and the fungus *Phytophthora cinnamomi* [27]. Violante et al. investigated the antibacterial activity of the extracts and fractions of *Roupala braziliensis*, revealing limited activity against *E. coli*, *E. faecalis*, *K. pneumoniae*, and *p. aeruginosa*, indicating a low or negligible capacity to inhibit the growth of these bacteria. However, activity was observed against *S. aureus* in all fractions, with MICs ranging between 15.6 and 125 µg/mL [26,28].

The reduced antibacterial properties of *R. montana* EO, when compared with findings on extracts from other species, might stem from a lower presence of major compounds with strong antibacterial properties. Despite its complex composition, the efficacy of an essential oil does not depend only on the presence of active compounds but also on the complex interactions among them [29]. It could be noted that *R. montana* EO lacks strong synergistic or additive effects. Antagonistic interactions, where some compounds diminish the effectiveness of other compounds, could explain the observed moderate activity [30]. Thus, EOs′ bioactivity reflects their entire chemical profile, not only dominant constituents.

*R. montana* EO exhibited a promising inhibitory effect, with an IC_50_ value of 23.27 ± 1.04 µg/mL, against AChE, which can be attributed to the main chemical compounds, without a clear distinction. However, since phytol is the major compound, its high abundance (21%) may explain the observed effect. This is supported by the study conducted by Sathya et al., in which phytol and phytol-loaded PLGA nanoparticles (phytol–PLGANPs), administered at a dose of 100 mg/kg, significantly reduced scopolamine-induced locomotor activity in rats. This effect was attributed to the potent inhibition of the rat brain acetylcholinesterase and butyrylcholinesterase enzymes [31]. To date, no studies have confirmed the acetylcholinesterase activity of essential oils from the genus *Roupala*. Our findings carry significant implications due to the promising activity identified. While it is acknowledged that numerous investigations have focused on alkaloids as the primary contributors to acetylcholinesterase activity [32], recent investigations have demonstrated that other classes of natural products, such as specific sesquiterpenes and diterpenes, can indeed inhibit AChE [33]. This is exemplified by the EO of *Jungia rugosa*, where chemical analysis of the plant revealed the presence of compounds exhibiting acetylcholinesterase inhibitory activity [34]. Our findings are significant because AChE inhibition represents a key mechanism in the therapeutic intervention of neurodegenerative diseases, such as Alzheimer’s disease [35].

Finally, the antioxidant capacity can be compared with that of *Oreocallis grandiflora* (Lam.) R. Br., a Proteaceae species. The leaf extract of *O. grandiflora* exhibited significant antiradical and anti-inflammatory effects in the DPPH assay (IC_50_ = 6.69 ± 1.39 µg/mL) [36]. Proteaceae species possess potential bioactive properties across diverse plant tissues [37,38]. Furthermore, studies focusing on the same species have generally presented varying results. This is exemplified by the ethanol extract of *O. grandiflora*, which demonstrated radical-scavenging capacity, with an IC_50_ value of 6.69 ± 1.39 µg/mL in the DPPH assay, while *O. grandiflora* flowers collected from the same location exhibited different radical-scavenging activity values, with IC_50_ values of 14.39 ± 1.43 µg/mL and 955.23 ± 0.25 µg/mL [36]. The antioxidant capacity of *H. terminalis* measured in the trunk showed an IC_50_ value of 156.9 mg/mL in the DPPH assay. Additionally, the aerial parts of *Roupala paulensis* have a total phenolic content of 24.27 ± 0.76 g GAE/100 g. Due to these results, Zhang et al. posit that the determination of antioxidant activity is influenced by the extraction solvents, plant origin, and analytical methods employed for quantification, suggesting the application of a wider range of antioxidant assays, such as ORAC and TEAC, alongside standardized extraction and analytical protocols, to achieve a more comprehensive and reliable understanding of the true antioxidant potential [37].

In brief, the biological properties of *R. montana* EO were demonstrated through various analyses; the results of this investigation open the possibility of identifying a promising EO candidate for diverse applications within the pharmaceutical and biotechnological sectors.

## 4. Materials and Methods

### 4.1. Materials and Chemical Reagents

A series of aliphatic hydrocarbons from C9 to C25 (Chem Service, Sigma-Aldrich, St. Louis, MO, USA) and helium as a gas carrier (INDURA, Quito, Ecuador) were used for the calibration curve in the gas chromatograph equipment coupled to a flame ionization detector (GC-FID). For the biological assays, several culture media were employed, including Mueller–Hinton II for bacteria and Sabouraud broth for fungi. Thioglycolate medium was employed to reactivate specifically the *Campylobacter jejuni* strain. All media were obtained from DIPCO (Quito, Ecuador). Antioxidants were revealed by the use of stable free radicals, such as 2′-azinobis-3-ethylbenzothiazoline-6-sulfonic acid (ABTS) and 2,2-diphenyl-1-picrylhydryl (DPPH), as well as Trolox as a positive control, all of them obtained from Sigma-Aldrich (St. Louis, MO, USA). A cholinesterase inhibition test was carried out with the inclusion of acetylcholinesterase from *Electrophorus electricus* (AChE), acetylthiocholine (ATCh), and Ellman’s reagent *(*DTNB). Solvents like cyclohexane and methanol were purchased from Sigma-Aldrich (St. Louis, MO, USA).

### 4.2. Plant Material

**Figure 5:**
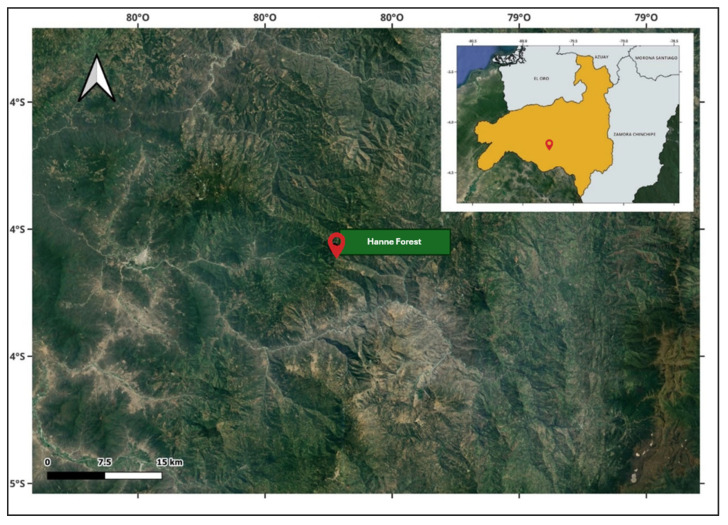
Geographic map showing sampling site for *Roupala montana* collection in Hanne Forest, southern Ecuador.

Leaves of *Roupala montana* were collected during the flowering period in Hanne Forest, Cariamanga cantón, at 2350 m a.s.l. (04°22′21″ S-79°43′05″ W) in the south of Ecuador (Figure 5). Our collections were conducted in accordance with Ecuadorian law and were authorized by the Ministry of Environment, Water, and Ecological Transition of Ecuador (MAATE) under permit code MAATE-DBI-CM-2022-0248. Upon collection, the plant was transported to the university facilities in airtight plastic containers. A specimen of the plant species was deposited in the Herbarium of the Universidad Técnica Particular de Loja (UTPL), under the voucher code 14814.

### 4.3. Post-Harvest Processing

The *Roupala montana* was dried using an electric dryer (model DY-330H, Lassele, Ansan City, Geyeonggi-do, Republic of Korea) for 72 h at 35 °C.

### 4.4. Isolation of Essential Oil

After drying, 100 g of the leaves of *R*. *montana* was subjected to hydro-distillation in a Clevenger-type apparatus with a return arm for aqueous distillate separation (purchased commercially) for 3 h from the collection of the first drop of distillate. Due to density differences, the EO was separated from the water by decantation. The EO was dried over anhydrous sodium sulfate to remove the moisture. The procedure was performed three times, and then the EO was stored at −4 °C.

### 4.5. Chemical Characterization of Essential Oil

#### 4.5.1. Sample Preparation

Gas chromatography (GC) analysis was carried out using analytical-grade solvents and *n*-nonane (Sigma-Aldrich, St. Louis, MO, USA) as the internal standard, following procedures well described in the literature [39]. Specifically, 10 µL of essential oil was diluted with 1 mL of an internal standard solution, prepared by dissolving 0.7 mg of *n*-nonane in cyclohexane to a final volume of 10 mL. The resulting solution was used for qualitative, quantitative, and enantiomeric analyses.

#### 4.5.2. Qualitative and Quantitative Analysis

The distilled EO from *Roupala montana* was analyzed using a gas chromatography–mass spectrometry (GC–MS) system comprising a Thermo Scientific (Waltham, MA, USA) Trace 1310 gas chromatograph paired with an AI/AS 1300 autosampler and an ISQ 7000 quadrupole mass detector. Data acquisition and processing were managed via the Chromeleon XPS software, version 7.2.10 (Waltham, MA, USA), and mass spectral matching was performed using the NIST Main Database (mainlib). Spectral acquisition was conducted in electron ionization (EI) mode at 70 eV, scanning within a mass range of 40–350 *m*/*z*. High-purity helium served as the carrier gas at a consistent flow rate of 1.0 mL/min. Each sample was analyzed by injecting 1 μL of the oil into a DB-5 ms capillary column (with a 30 m × 0.25 mm internal diameter and a 0.25 μm film thickness, coated with 5% phenylmethylpolysiloxane). The column oven was initially held at 60 °C for 5 min, then ramped up to 200 °C at 2 °C/min, followed by a final increase to 250 °C at 15 °C/min, and maintained for an additional 5 min. The ion source and quadrupole chamber temperatures were set at 230 °C and 150 °C, respectively. All chromatographic runs were conducted in triplicate to ensure reproducibility.

To quantify the volatile constituents, GC coupled with a flame ionization detector (GC–FID) was performed using the same stationary phase and operational parameters as the GC–MS analysis. The split injection mode was used with a ratio of 1:40 to ensure an optimal peak resolution and detector sensitivity.

The compound identification was based on the comparison of both their mass fragmentation patterns and linear retention indices (LRIs) with previously published reference data [10]. The LRI of each analyte was calculated using the Van Den Dool and Kratz formula [40], employing a homologous series of *n*-alkanes run under identical experimental conditions. Equation (1) estimates the LRI using the retention times of the bracketing *n*-alkanes and that of the target analyte, allowing for improved reliability in compound annotation, where RI represents the retention index, C represents the C_9_ to C_25_ aliphatic homologous series, RTx represents the retention time of the analyzed compound, RTn represents the retention time of the aliphatic hydrocarbon that was eluted before, and RTN represents the retention time of the aliphatic hydrocarbon that was eluted before.

(1) $$RI=100C+100\frac{\left(RTx-RTn\right)}{\left(RTN-RTn\right)}$$

#### 4.5.3. Enantiomeric Analysis

Enantiomeric analysis of the *R. montana* EO was performed with the previous sample preparation using quantitative and qualitative analysis and the same GC–EM system. However, the oven program was held at 60 °C for 5 min and then increased at a rate of 2 °C to 220 °C for 2 min, using a chiral capillary column, MEGA-DEX-DET-Beta, with diethyl-tert-butylsilyl-β-cyclodextrin (25 m × 0.25 mm × 0.25 μm) (Mega, Legnano, Italy). The injection of a series of homologous C_9_ to C_24_ alkanes enabled the calculation of the linear retention indices of the stereoisomers. The enantiomeric excess was determined by the calculated difference between the percentage of the major enantiomer and that of the minor enantiomer.

### 4.6. Biological Activities

#### 4.6.1. Antimicrobial Activity

The assay was conducted using a modified procedure adapted from Cartuche et al.’s study [41]. The minimum inhibitory concentration (MIC) was determined using the broth microdilution method to evaluate the effect of the essential oil against nine ATCC reference strains. The panel of microorganisms comprised three Gram-positive cocci (*Enterococcus faecalis* (ATCC^®^ 19433), *Enterococcus faecium* (ATCC 27270), and *Staphylococcus aureus* (ATCC 25923)), one Gram-positive Bacillus (*Lysteria monocytogenes* (ATTC^®^ 19115)), three Gram-negative bacilli (*Escherichia coli* (O157:H7) (ATCC^®^ 43888), *Campylobacter jejuni* (ATCC^®^ 33560), and *Salmonella enterica* (ATCC^®^ 14028)), and finally, two molds (*Candida albicans* (ATCC^®^ 10231) and *Aspergillus niger* (ATCC^®^ 6275)).

The EO was initially diluted to 80 mg/mL in dimethyl sulfoxide (DMSO), and subsequently, the two-fold serial dilution method was performed to achieve a final concentration ranging from 4000 to 31.25 μg/mL The final cell concentrations in the assay wells were 5 × 10^5^ colony-forming units (CFUs)/mL for bacteria, 2.5 × 10^5^ CFUs/mL for yeast, and 5 × 10^4^ spores/mL for the sporulated fungus. The assay was conducted in 96-well microplates using Mueller–Hinton II (MH II) broth for bacteria and Sabouraud dextrose broth for fungi as the growth media. Commercial antimicrobial agents, including ampicillin (1 mg/mL solution) for *S. aureus*, *E. faecalis*, and *E. faecium*, ciprofloxacin (1 mg/mL solution) for *L. monocytogenes*, *E. coli*, and *S. enterica*, erythromycin (1 mg/mL solution) for *C. jejuni*, and amphotericin B (250 µg/mL) for the tested fungi, served as positive controls. DMSO was used as the negative control at a maximum final concentration of 5%.

#### 4.6.2. Anticholinesterase Activity

The AChE inhibition was measured using the method developed by Andrade et al. [42]. Acetylcholinesterase inhibition was assessed following the addition of acetylthiocholine as the enzymatic substrate and varying concentrations of the essential oil dissolved in methanol. Briefly, the reaction mixture contained 40 μL of Tris buffer (pH 8.0), 20 μL of the EO sample, 20 μL of acetylthiocholine (ATCh at 15 mM in PBS (pH 7.4)), and 100 μL of DTNB as a reaction revealer (at 3 mM in Tris buffer). A 3 min preincubation at 25 °C with continuous shaking was followed by the addition of 20 μL of the enzyme acetylcholinesterase (at 0.5 U/mL). The progression of the enzymatic reaction was monitored spectrophotometrically at 405 nm for 60 min using a microplate reader (EPOCH 2, BioTek, Winooski, VT, USA). To determine enzyme inhibition, the EO was tested at final concentrations of 1000, 500, 100, 50, and 10 µg/mL in methanol. The assay was performed in triplicate using 96-well microplates. Donepezil served as a positive control. IC_50_ values were subsequently calculated from the kinetic progression curves using the GraphPad Prism software (version 8.0.1).

#### 4.6.3. Antioxidant Activity

##### ABTS

The antioxidant capacity against the ABTS^•+^ cation (2,2′-azinobis-3-ethylbenzothiazoline-6-sulfonic acid) was determined following the methods reported by Valarezo et al. [43]. The EO was initially diluted in methanol to a concentration of 80 mg/mL. A two-fold serial dilution was then performed to obtain seven consecutive dilutions, resulting in final EO concentrations ranging from 8000 to 62.5 µg/mL. A stock solution of ABTS^•+^ radicals was generated by reacting equal volumes of aqueous ABTS (7.4 µM) and potassium persulfate (2.6 µM) solutions under constant stirring for 14 h. A working radical solution was subsequently prepared by diluting an aliquot of the stock solution with methanol until an absorbance of 1.1 ± 0.02 was reached. Measurements were performed at 734 nm using an EPOCH 2 microplate reader (BioTek, Winooski, VT, USA). Antiradical activity was assessed by reacting 270 µL of the ABTS^•+^ working adjusted solution with 30 µL of the essential oil dilutions. The absorbance was monitored at 734 nm in darkness at room temperature for 60 min. Trolox and methanol served as positive and blank controls, respectively. The results are expressed as SC_50_ values (the 50% radical scavenging concentration), calculated from the concentration–response curves using the GraphPad Prism software (version 8.0.1). All measurements were performed in triplicate. A Trolox calibration curve was established using concentrations ranging from 1.25 to 50 µM, employing the same assay procedure.

##### DPPH

The DPPH assay was conducted following the procedure described by Valarezo et al. [44]. The highest concentration evaluated was 8,000 µg/mL. The EO was prepared as described above for the ABTS technique. The equipment, negative control, and positive control were identical to those employed in the ABTS assay, with the key difference being that the antiradical capacity of the essential oil of *R. montana* was determined by measuring the reduction in DPPH^•^ absorbance at 515 nm. The SC_50_ value was calculated as described above.

### 4.7. Statistical Processing

The data were obtained and processed using Microsoft Excel. Rstudio (R version 4.4.2, R Foundation for Statistical Computing, Vienna, Austria) was used to determine the measures of central tendency and standard deviation. The data of the anticholinesterase activity and antioxidant capacity assays were analyzed with GraphPad Prism (version 8.0.1, GraphPad Software Inc., San Diego, CA, USA). The IC_50_ value for the AChE assay was calculated by curve fitting, non-linear regression analysis, and the log inhibitor vs. normalized response–variable slope model. The isolation of the essential oil of *R. montana*, antioxidant capacity evaluation, and anticholinesterase activity analysis were performed with three replicates, as well as the chromatographic assays. The enantiomeric composition was determined from a single chromatographic run. As the analysis was not replicated, standard deviation values are not reported.

## 5. Conclusions

To the best of our knowledge, this study represents the first comprehensive phytochemical and biological evaluation of the essential oil (EO) from *Roupala montana* leaves from Ecuador. The EO exhibited a complex volatile composition dominated by diterpenes, with phytol as the major constituent. Enantioselective analysis revealed high enantiomeric excess for (S)-(+)-γ-muurolene and complete purity for (1S,4aR,8aR)-(−)-γ-cadinene, emphasizing the stereochemical uniqueness of the oil. Antimicrobial assays demonstrated moderate antifungal activity against *Aspergillus niger* and selective antibacterial activity against *Campylobacter jejuni*, as well as a promising acetylcholinesterase (AChE) inhibitory effect (IC_50_ = 23.27 ± 1.04 µg/mL), potentially associated with its high phytol content. In contrast, the EO exhibited negligible antioxidant activity in both ABTS and DPPH assays. Taken together, these findings highlight the potential of *R. montana* EO, particularly for its neuroprotective implications via AChE inhibition, while also expanding the chemotaxonomic and phytochemical knowledge of the Proteaceae family in Ecuador.

## Figures and Tables

**Table 1:** Gas chromatography analysis of *Roupala montana* EO.

No.	t_RT_	Compound	Molecular Formula	LRI^a^	LRI^b^	% ± SD
1	25.17	*n*-decanol	C_10_H_22_O	1274	1266	0.62 ± 0.03
2	31.86	*β*-duprezianene	C_15_H_24_	1426	1421	3.16 ± 0.16
3	33.12	(2*E*)-dodecenal	C_12_H_22_O	1457	1464	0.35 ± 0.02
4	33.41	*α*-humulene	C_15_H_24_	1463	1452	0.70 ± 0.04
5	33.58	9-epi-(*E*)-caryophyllene	C_15_H_24_	1467	1464	1.31 ± 0.06
6	34.84	*α*-muurolene	C_15_H_24_	1498	1500	0.68 ± 0.04
7	35.04	*n*-pentadecane	C_15_H_32_	1503	1500	0.22 ± 0.01
8	35.94	*δ*-cadinene	C_15_H_24_	1525	1522	0.42 ± 0.02
9	37.62	(*E*)-nerolidol	C_15_H_26_O	1568	1561	1.67 ± 0.07
10	37.96	(*Z*)-dihydro-apofarnesol	C_14_H_26_O	1576	1571	2.52 ± 0.10
11	38.76	caryophyllene oxide	C_15_H_24_O	1597	1582	0.34 ± 0.02
12	38.87	*n*-hexadecane	C_16_H_32_	1600	1600	1.20 ± 0.05
13	39.26	2,(7*Z*)-bisaboladien-4-ol	C_15_H_26_O	1610	1618	0.90 ± 0.04
14	39.62	cis-isolongifolanone	C_15_H_24_O	1620	1612	0.47 ± 0.02
15	39.86	junenol	C_15_H_26_O	1626	1618	0.41 ± 0.02
16	40.38	cubenol	C_15_H_26_O	1640	1645	0.59 ± 0.03
17	41.81	*n*-tetradecanol	C_14_H_30_O	1678	1671	0.62 ± 0.03
18	42.01	elemol acetate	C_17_H_28_O_2_	1683	1680	tr
19	42.12	4-cuprenen-1-ol	C_15_H_24_O	1686	1692	0.40 ± 0.01
20	42.55	*n*-heptadecane	C_17_H_36_	1698	1700	0.25 ± 0.01
21	42.84	2-pentadecanone	C_15_H_30_O	1705	1697	0.79 ± 0.03
22	43.48	(2Z,6*E*)-farnesol	C_15_H_26_O	1724	1722	0.71 ± 0.03
23	43.58	(2*E*,6Z)-farnesol	C_15_H_26_O	1726	1714	tr
24	43.71	methyl tetradecanoate	C_15_H_30_O_2_	1730	1722	0.75 ± 0.02
25	44.63	(2*E*,6*E*)-farnesol	C_15_H_26_O	1756	1742	0.55 ± 0.02
26	45.31	butyl dodecanoate	C_16_H_32_O_2_	1775	1786	1.72 ± 0.09
27	45.93	*β*-eudesmol acetate	C_17_H_28_O_2_	1792	1792	0.08 ± 0.01
28	46.11	*n*-octadecane	C_18_H_38_	1797	1800	0.67 ± 0.03
29	47.77	hexahydrofarnesyl acetone	C_18_H_36_O	1843	1843	6.28 ± 0.26
30	48.77	cis-thujopsenic acid	C_15_H_22_O_2_	1871	1863	0.23 ± 0.00
31	49.24	*n*-hexadecanol	C_16_H_34_O	1889	1874	0.80 ± 0.02
32	49.50	*n*-nonadecane	C_19_H_40_	1897	1900	0.42 ± 0.02
33	50.21	(*5E-*9*E*)-farnesyl acetone	C_18_H_30_O	1919	1913	1.25 ± 0.03
34	50.60	methyl hexadecanoate	C_17_H_34_O_2_	1931	1921	2.96 ± 0.12
35	51.14	isophytol	C_20_H_40_O	1948	1946	1.29 ± 0.00
36	51.24	(*Z*,*Z*)-geranyl linalool	C_20_H_43_O	1951	1960	0.69 ± 0.01
37	52.12	hexadecanoic acid	C_16_H_32_O_2_	1978	1959	8.30 ± 0.77
38	52.76	ethyl hexadecanoate	C_18_H_36_O_2_	1997	1992	1.86 ± 1.41
39	53.57	1-eicosene	C_20_H_40_	2024	1988	0.13 ± 0.00
40	53.74	(*E*,*E*)-geranyl linalool	C_20_H_34_O	2029	2026	0.53 ± 0.02
41	55.93	*n*-heneicosane	C_21_H_44_	2100	2100	2.00 ± 0.00
42	56.14	laurenan-2-one	C_20_H_32_O	2107	2115	2.68 ± 0.03
43	56.26	NI	-	2111	-	tr
44	56.40	phytol	C_20_H_40_O	2116	2116	21.17 ± 1.59
45	56.87	incensole	C_20_H_34_O_2_	2132	2158	tr
46	57.35	1-docosene	C_22_H_44_	2148	2189	0.71 ± 0.40
47	57.47	nezukol	C_20_H_34_O	2152	2132	0.61 ± 0.79
48	57.68	linoleic acid	C_18_H_32_O_2_	2159	2132	1.09 ± 1.20
49	57.91	sandaracopimarinal	C_20_H_30_O	2167	2189	1.09 ± 0.76
50	58.11	ugandensidial (cinnamodial)	C_17_H_24_O_5_	2173	2198	1.80 ± 1.22
51	58.53	incensole acetate	C_22_H_36_O_3_	2188	2184	0.40 ± 0.28
52	58.68	oleic acid	C_18_H_34_O_2_	2193	2175	0.63 ± 0.12
53	58.80	*n*-docosane	C_22_H_46_	2197	2200	1.25 ± 0.55
54	59.35	7-*α*-hydroxy-manool	C_20_H_34_O_2_	2215	2237	1.31 ± 1.50
55	61.63	*n*-tricosane	C_23_H_48_	2290	2300	2.16 ± 0.00
56	63.46	3-*α*-acetoxy-manool	C_22_H_36_O_3_	2350	2359	0.43 ± 0.44
57	64.51	methyl labdanolate	C_21_H_38_O_3_	2385	2381	2.46 ± 0.01
58	64.63	*n*-tetracosane	C_24_H_50_	2389	2400	1.66 ± 0.01
59	66.13	NI	-	2438	-	2.26 ± 0.90
60	68.42	*n*-pentacosane	C_25_H_52_	2514	2500	9.08 ± 0.04
						
		Oxygenated monoterpenes (%)				0.62
		Oxygenated sesquiterpenes (%)				7.81
		Sesquiterpenes hydrocarbons (%)				6.49
		Diterpene hydrocarbons				0.13
		Oxygenated diterpenes (%)				29.37
		Other compounds (%)				55.21
		Total (%)				99.63

LRI^a^, calculated linear retention index; LRI^b^, linear retention index from the literature [10]; **t_RT_**, retention time; %, percentage; SD, standard deviation; tr, traces. Both values are conveyed as means of three determinations. NI. Non identified.

**Table 2:** Chiral compounds present in *R*. *montana* leaf essential oil.

Enantiomer	LRI^a^	LRI^b^	ED (%)	e.e. (%)
(S)-(+)-γ-muurolene	1471	1466	97.53	95.07
(R)-(−) γ-muurolene	1474	1473	2.47	
(1S,4aR,8aR)-(−)-γ -cadinene	1529	1526	100.00	100.00

LRI^a^, calculated linear retention index; LRI^b^ [11,12]; ED: enantiomeric distribution; e.e. (%), percent enantiomeric excess.

**Table 3:** Antifungal activity of *Roupala montana* leaf essential oil.

Microorganism	*Roupala montana*	Positive Control *	Negative Control
	MIC (µg/mL)		
Yeasts			
*Candida albicans* (ATTC 10231)	-	0.098	+
Fungi			
*Aspergillus niger* (ATCC 6275)	1000	0.098	+

*: amphotericin B; MIC: minimum inhibitory concentration; +: normal growth at 5% DMSO.

**Table 4:** Antibacterial capacity of *Roupala montana* essential oil.

Microorganism	*Roupala montana*	Positive Control *	Negative Control
	MIC (µg/mL)		
Gram-positive cocci			
*Enterococcus faecalis* (ATCC 19433)	-	0.78	+
*Enterococcus faecium* (ATCC 27270)	-	0.39	+
*Staphylococcus aureus* (ATCC 25923)	-	0.39	+
Gram-positive bacillus			
*Lysteria monocytogenes* (ATTC 19115)	-	1.56	+
Gram-negative bacilli			
*Escherichia coli* O157:H7 (ATCC 43888)	-	1.5600	+
*Campylobacter jejuni* (ATCC 33560)	500	15.65	+
*Salmonella enterica* subs enterica serovar Thypimurium WDCM 00031, derived (ATCC 14028)	-	0.39	+

*: ampicillin for Gram-positive cocci, and erythromycin (*Campylobacter jejuni*) and ciprofloxacin for Gram-negative bacilli and Gram-positive bacillus; +: normal growth.

**Table 5:** Radical-scavenging capacity of EO.

EO	ABTS	DPPH
	SC_50_ (µg/mL—µM *) ± SD	
*R. montana*	>8000	-
Trolox	29.09 ± 1.05	35.54 ± 1.04

* Half scavenging capacity of Trolox is expressed in micromolar units.

## Data Availability

All data presented in this study are available in this article.

## Supplementary Materials

The following supporting information can be downloaded at: https://www.mdpi.com/article/10.3390/molecules30163323/s1, Figure S1: Complete gas chromatogram of *Roupala montana* leaves essential oil. The peak at approximately 8.15 min corresponds to the internal standard, n-Nonane, used in the analysis.; Figure S2: Enantioselective analysis of *Roupala montana* EO on a 2,3-diethyl-6-tert-butyldimethylsilyl-β-cyclodextrin stationary phase. The peak at approximately 2.86 min corresponds to the internal standard, n-Nonane, used in the analysis.
