# Supplementary material for: Roupala montana Aubl. Essential Oil: Chemical Composition and Emerging Biological Activities
Source: Molecules. 2025 Aug 8;30(16):3323. doi: 10.3390/molecules30163323 (PMC12388818; doi:10.3390/molecules30163323)
Supplement: Supplementary file 1 [file molecules-30-03323-s001.zip › molecules-3776790-supplementary.pdf]

SUPPLEMENTARY MATERIAL

Roupala montana Aubl. Essential Oil: Chemical Composition and Emerging Biological Activities

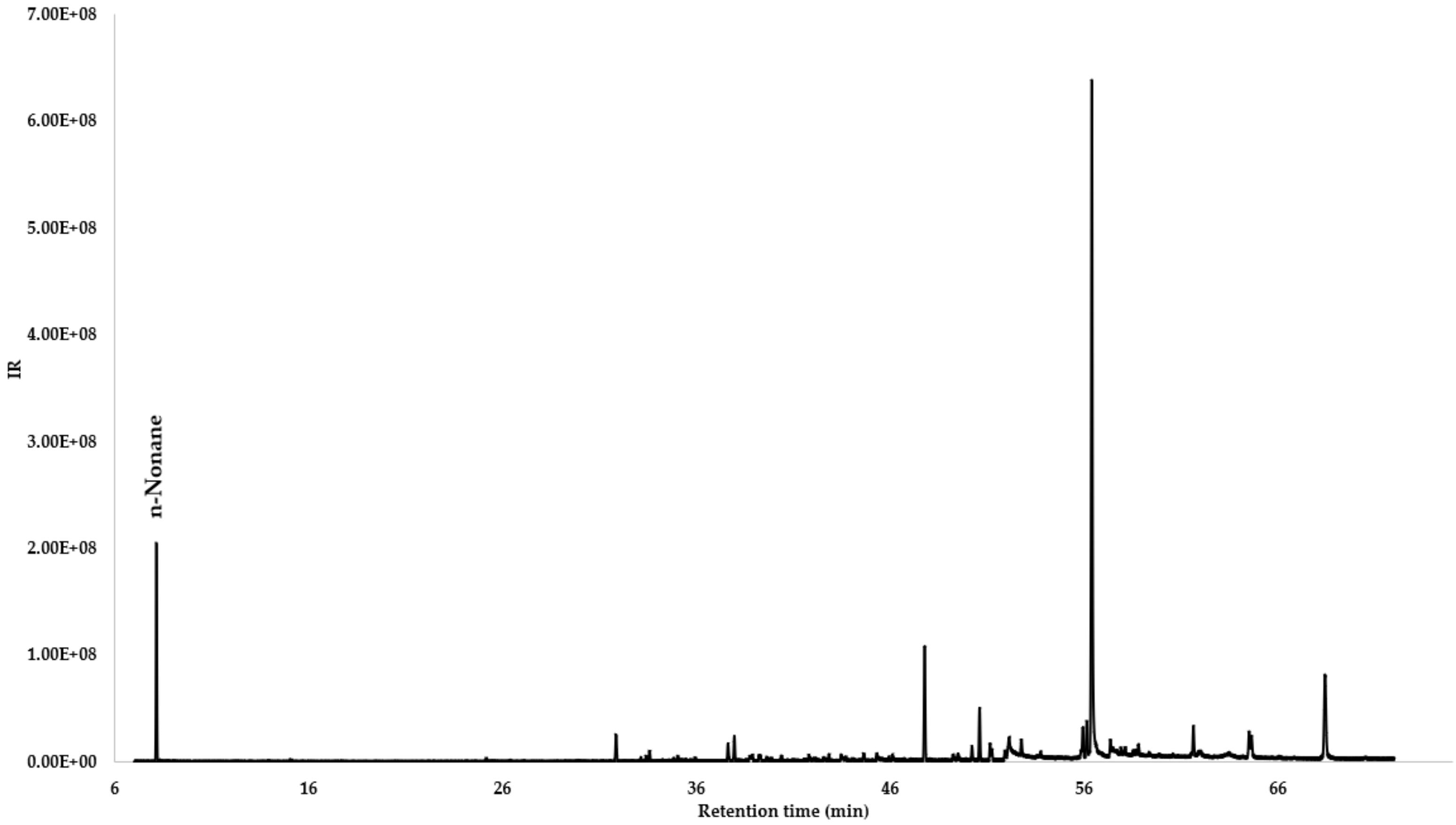

Figure S1. Complete gas chromatogram of *Roupala montana* leaves essential oil. The peak at approximately 8.15 minutes corresponds to the internal standard, n-Nonane, used in the analysis.

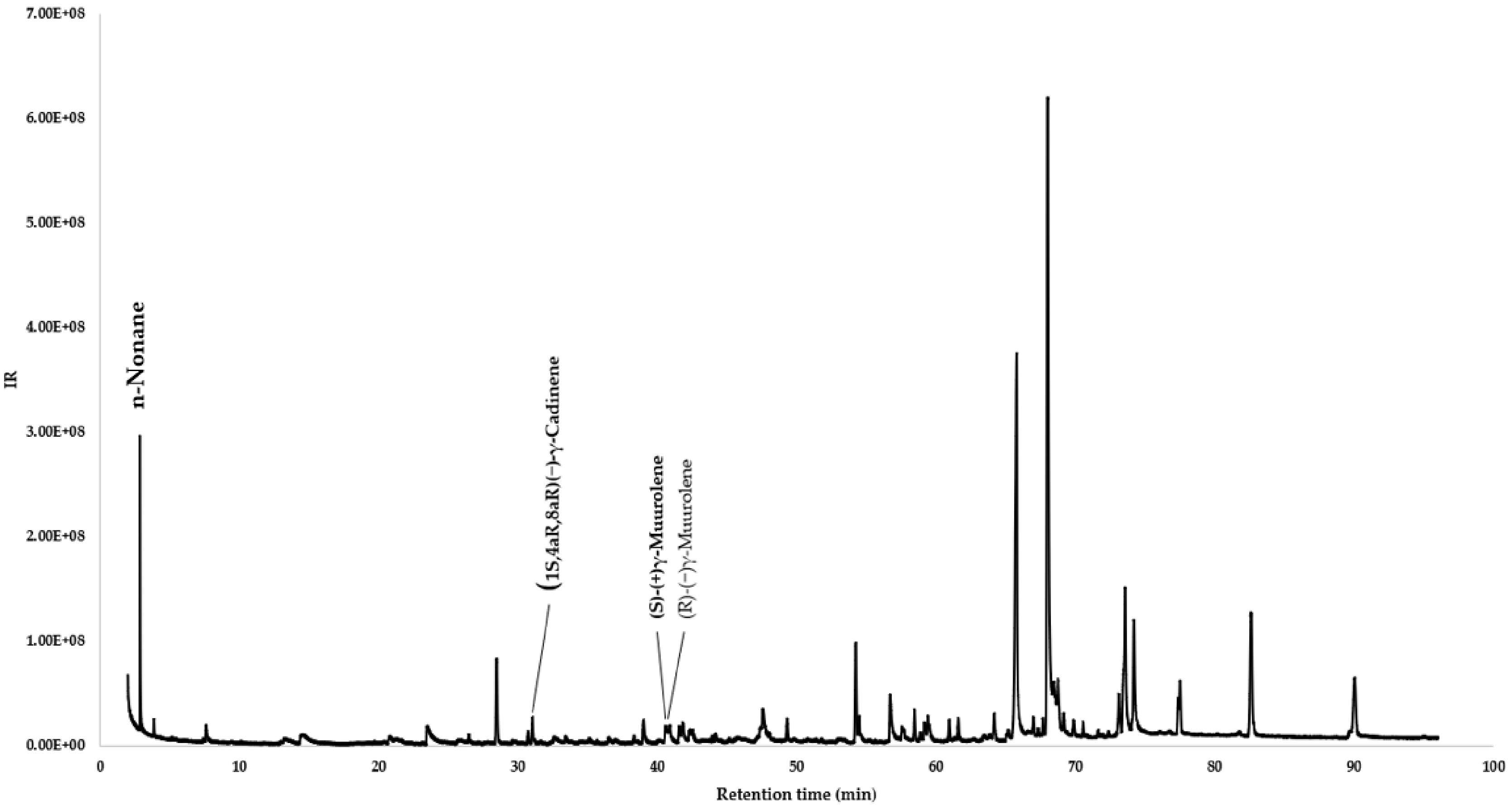

Figure S2. Enantioselective analysis of *Roupala montana* EO on a 2,3-diethyl-6-*tert*-butyldimethylsilyl-β-cyclodextrin stationary phase. The peak at approximately 2.86 minutes corresponds to the internal standard, n-Nonane, used in the analysis.
